# Effects of Tert-Butylhydroquinone on Intestinal Inflammatory Response and Apoptosis following Traumatic Brain Injury in Mice

**DOI:** 10.1155/2010/502564

**Published:** 2011-01-11

**Authors:** Wei Jin, Hongbin Ni, Yuxiang Dai, Handong Wang, Tianyu Lu, Jun Wu, Jian Jiang, Weibang Liang

**Affiliations:** ^1^Department of Neurosurgery, Drum Tower Hospital, Medical School of Nanjing University, 321 Zhongshan Road, Nanjing, Jiangsu 210008, China; ^2^Department of Neurosurgery, Jinling Hospital, Medical School of Nanjing University, Nanjing, Jiangsu 210002, China

## Abstract

Traumatic brain injury (TBI) can induce intestinal inflammatory response and mucosal injury. Antioxidant transcription factor nuclear factor erythroid 2-related factor 2 (Nrf2) has been shown in our previous studies to prevent oxidative stress and inflammatory response in gut after TBI. The objective of this study was to test whether tert-butylhydroquinone (tBHQ), an Nrf2 inducer, can protect against TBI-induced intestinal inflammatory response and mucosal injury in mice. Adult male ICR mice were randomly divided into three groups: (1) sham + vehicle group, (2) TBI + vehicle group, and (3) TBI + tBHQ group (*n* = 12 per group). Closed head injury was adopted using Hall's weight-dropping method. Intestinal mucosa apoptosis and inflammatory-related factors, such as nuclear factor kappa B (NF-*κ*B), tumor necrosis factor-*α* (TNF-*α*), interleukin-1*β* (IL-1*β*), interleukin-6 (IL-6) and intercellular adhesion molecule-1 (ICAM-1), were investigated at 24 h after TBI. As a result, we found that oral treatment with 1% tBHQ prior to TBI for one week markedly decreased NF-*κ*B activation, inflammatory cytokines production, and ICAM-1 expression in the gut. Administration of tBHQ also significantly attenuated TBI-induced intestinal mucosal apoptosis. The results of the present study suggest that tBHQ administration could suppress the intestinal inflammation and reduce the mucosal damage following TBI.

## 1. Introduction

Traumatic brain injury (TBI) is known to cause several secondary effects, which lead to peripheral organs dysfunction. The close relationship between brain injury and subsequent intestinal mucosal injury has been recognized [1, 2]. This pathologic course may not only influence the intestinal mucosa itself but also impair the remote organs, leading to systemic inflammatory response syndrome (SIRS) and multiple-organ dysfunction syndrome (MODS) [3]. Previous studies of our laboratory demonstrated that traumatic brain injury could lead to intestinal inflammatory response mediated by increased nuclear factor kappa B (NF-*κ*B) and proinflammatory cytokines, which played a critical role in the pathogenesis of acute gut mucosal injury following TBI [4, 5]. Afterwards, using nuclear factor erythroid 2-related factor 2 (Nrf2) knockout mice, we found that Nrf2, which is the key transcription factor that mediates the induction of cellular antioxidant defense mechanisms, played an important protective role in limiting TBI-induced intestinal inflammatory response and gut mucosal injury [6, 7]. However, it is not known whether activation of the factor Nrf2 has a protective effect in the intestinal inflammation associated with TBI. 

A wide range of natural and synthetic small molecules with diverse chemical backgrounds have been shown to induce the Nrf2 activity; tert-butylhydroquione (tBHQ) is the major one that exerts powerful antioxidant potential [8–10]. It has been documented that tBHQ protects the animals and cell lines against acute toxicity and oxidative insult, presumably through the induction of many cytoprotective and detoxifying enzymes such as epoxide hydrolase [11], glutathione-S-transferase [12], and glucoronyltransferase [13]. This activation is dependent on the translocation of Nrf2 from the cytoplasm to the nucleus through inducer interaction with Keap1, the cytoplasmic repressor of Nrf2 [14]. Nevertheless till now, no study was found in the literature to address the therapeutic effect of the activation of Nrf2 by tBHQ on the TBI-induced intestinal inflammatory response. The purpose of the current study was therefore to determine the influence of tBHQ administration on intestinal NF-*κ*B activity, proinflammatory cytokines expression, and apoptosis after TBI.

## 2. Materials and Methods

### 2.1. Animals

All procedures were approved by the Institutional Animal Care Committee and were in accordance with the guidelines of the National Institutes of Health on the care and use of animals. Age- and weight-matched adult male ICR mice (6–8 weeks, 28–32 g) were purchased from Animal Center of Chinese Academy of Sciences, Shanghai, China. Mice were housed at 23 ± 1°C in humidity-controlled animal quarters with 12-hour light/dark cycle.

### 2.2. Experimental Protocol

The experimental groups consisted of sham + vehicle group, TBI + vehicle group, and TBI + tBHQ group (*n* = 12 per group). Mice of TBI + tBHQ group were fed the tBHQ-supplemented diet for one week prior to injury, but mice of sham + vehicle group and TBI + vehicle group were given the control food. For tBHQ feeding, food pellets were powdered in a coffee grinder and dry mixed with 1% tBHQ (w/w). Distilled water was added to the powder (equal v/w), thoroughly mixed, and reshaped into food pellets. The pellets were then baked at 60°C for 3 h. Control food was processed in the same manner without the addition of tBHQ [15]. This dose and medication of tBHQ administration have been widely used in analogous animal models described elsewhere [15, 16].

The mouse model of TBI was employed as described [17] being previously developed in our laboratory [18]. Mice were anesthetized by intraperitoneal injection with sodium pentobarbital (50 mg/kg). A round, flat, and 6 mm diameter Teflon impounder was centered between the ears and eyes. TBI was induced by a 100 g weight dropped from a 12 cm height along a stainless steel string, which translated into 1200 g/cm. This model is generally associated with 20% mortality within the first 5 min after injury, and no delayer mortality was observed thereafter [18]. Sham mice were subjected to identical treatment with the exception that no injury was performed. 

Animals were decapitated at 24 h following sham or injury for sample collection. Six mice in each group were sacrificed for electrophoretic mobility shift assay (EMSA) and enzyme-linked immunosorbent assay (ELISA), the others were for immunohistochemistry and terminal deoxynucleotidyl transferase-mediated dUTP nick end labeling (TUNEL) study. For EMSA and ELISA analysis, mice were exsanguinated by cardiac puncture, and a 3-cm segment of the jejunum 8 cm distal to Treitz ligament was rapidly removed and stored at liquid nitrogen immediately. For immunohistochemistry and TUNEL study, mice were perfused via left ventricular puncture with cold saline (4°C), followed by 4% neutral buffered formalin. The 3-cm segment of the jejunum was taken, stored overnight in 4% neutral buffered formalin, and then embedded in paraffin.

### 2.3. Nuclear Protein Extract and EMSA

The DNA binding activity of NF-*κ*B in intestine was determined by EMSA. Nuclear protein of intestinal tissue was extracted and quantified as described previously in [6]. EMSA was performed using a commercial kit (Gel Shift Assay System; Promega, Madison, WI) following the methods in our laboratory. A Consensus oligonucleotide probe for NF-*κ*B (5′-AGT TGA GGG GAC TTT CCC AGG C-3′) was end-labeled with [*γ*-^32^P]-ATP (Free Biotech., Beijing, China) with T4-polynucleotide kinase. EMSA was performed according to our previous study [6]. NF-*κ*B activity was quantified by computer-assisted densitometric analysis.

### 2.4. ELISA Analysis

The intestinal levels of inflammatory cytokines such as tumor necrosis factor-*α* (TNF-*α*), interleukin-1*β* (IL-1*β*), and interleukin-6 (IL-6) were quantified using ELISA kits specific for mouse according to the manufacturers' instructions (TNF-*α* from Diaclone Research, France; IL-1*β*, IL-6 from Biosource Europe SA, Belgium) and our previous study [6]. The cytokine contents in the intestinal tissue were expressed as pg per milligram protein.

### 2.5. Immunohistochemical Staining

The paraffin-embedded sections (4 *μ*m) were used for Immunohistochemical assay, which was performed with a goat antimouse intercellular adhesion molecule-1 (ICAM-1) antibody (diluted 1 : 200, R&D Systems, Inc., Minnesota, USA), according to our previous study [6]. Microscopy of the immunohistochemically stained tissue sections was performed by an experienced pathologist blinded to the experimental condition. Evaluation of sections was undertaken by assessing the intensity of staining (5 grades). “0” indicates no detectable positive cell; “1” indicates very low density of positive cells, “2” indicates a moderate density of positive cells, “3” indicates the higher, but not maximal density of positive cells, and “4” indicates the highest density of positive cells. 

### 2.6. TUNEL Study

The paraffin-embedded sections were also detected for apoptotic cells by TUNEL method. The procedures were according to instruction of the kit (ISCDD, Boehringer Mannheim, Germany) and our laboratory methods [7]. Ten villi and crypts of each section were observed under a light microscope, and the average number of TUNEL-positive cells in 100 counted cells was assigned as the apoptotic index. The distinctive morphological features of apoptosis were used to recognize apoptotic cells. Small clusters of dead cell fragments were assessed as originating from one cell and given a single count, and any doubtful cells were disregarded.

### 2.7. Statistical Analysis

Software SPSS 13.0 was used for the statistical analysis. All data were expressed as mean ± SEM. The measurements were subjected to one-way ANOVA. Differences between experimental groups were determined by Fisher's LSD posttest. Significance was assigned at *P* < .05.

## 3. Results

### 3.1. EMSA for NF-*κ*B

**Figure 1:**
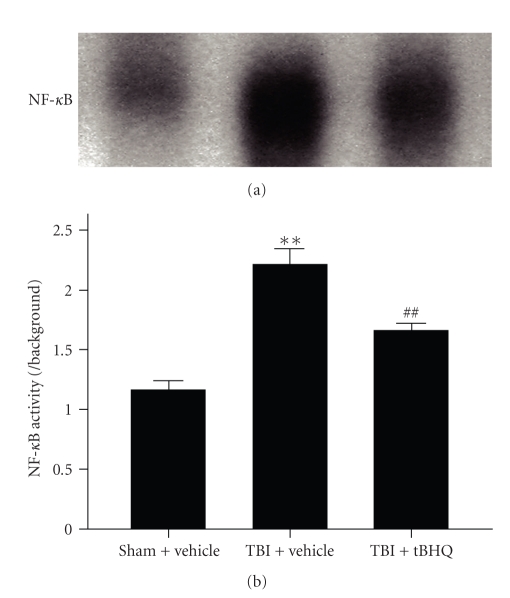
NF-*κ*B binding activity in the intestinal tissue in sham + vehicle group, TBI + vehicle group, and TBI + tBHQ group (*n* = 6). EMSA autoradiography of NF-*κ*B DNA binding is shown on top of the graph, and the order of individual bands corresponds to that of graph bars. Lane 1: sham + vehicle group; lane 2: TBI + vehicle group; lane 3: TBI + tBHQ group. tBHQ treatment markedly suppressed the activation of NF-*κ*B in the gut following TBI. **P < .01 versus sham + vehicle group; ^##^ *P* < .01 versus TBI + vehicle group.

As shown in Figure 1, low intestinal NF-*κ*B binding activity (weak EMSA autoradiography) was detected in the sham + vehicle group. Compared with sham + vehicle group, NF-*κ*B binding activity in the intestinal tissue was significantly increased in TBI + vehicle group. In TBI + tBHQ group, the intestinal Nrf2 binding activity was significantly downregulated after TBI. The results showed that tBHQ treatment could markedly suppress the activation of NF-*κ*B in the gut following TBI.

### 3.2. ELISA for Inflammatory Cytokines

**Figure 2:**
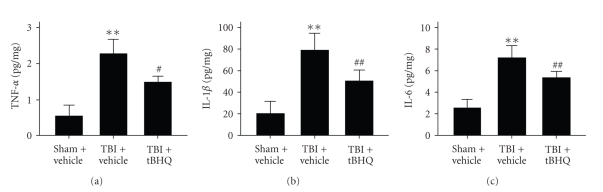
Concentrations of inflammatory cytokines in the intestinal tissue in sham + vehicle group, TBI + vehicle group, and TBI + tBHQ group (*n* = 6). The figure indicates that concentrations of TNF-*α*, IL-1*β*, and IL-6 in the intestine were significantly increased after TBI and could be suppressed when treated with tBHQ. **P < .01 versus sham + vehicle group. ^#^ *P* < .05 and ^##^ *P* < .01 versus TBI + vehicle group.

Concentrations of inflammatory cytokines including TNF-*α*, IL-1*β*, and IL-6 were low in the mice gut of sham + vehicle group, as shown in Figure 2. Compared with sham + vehicle group, intestinal levels of TNF-*α*, IL-1*β*, and IL-6 were greatly induced in TBI + vehicle group. tBHQ administration after TBI could lead to significantly decreased TNF-*α*, IL-1*β*, and IL-6 concentrations in mice intestinal tissue. 

### 3.3. Immunohistochemistry for ICAM-1

**Figure 3:**
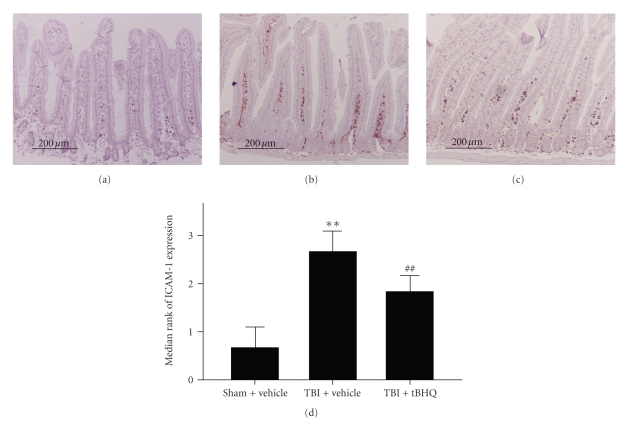
Expression of ICAM-1 in the intestinal tissue in sham + vehicle group, TBI + vehicle group, and TBI + tBHQ group (*n* = 6). (a) Sham + vehicle group mice showing low ICAM-1 immunoreactivity. (b) TBI + vehicle group showing increased ICAM-1 immunoreactivity. (c) TBI + tBHQ group mice showing less ICAM-1 immunoreactivity than those of TBI + vehicle group. (d) tBHQ administration significantly downregulated the ICAM-1 expression in mice gut following TBI. **P < .01 versus sham + vehicle group; ^##^ *P* < .01 versus TBI + vehicle group.

The immunohistochemical assay showed low ICAM-1 immunoreactivity in the villous interstitium and lamina propria in sham + vehicle group; see Figure 3. Compared to the sham + vehicle group, ICAM-1 in the intestinal tissue was significantly upregulated in TBI + vehicle group. In TBI + tBHQ group, the level of ICAM-1 immunoreactivity was significantly decreased. The results suggested that tBHQ administration could significantly down-regulate the ICAM-1 expression in mice gut following TBI.

### 3.4. TUNEL for Intestinal Mucosal Apoptosis

**Figure 4:**
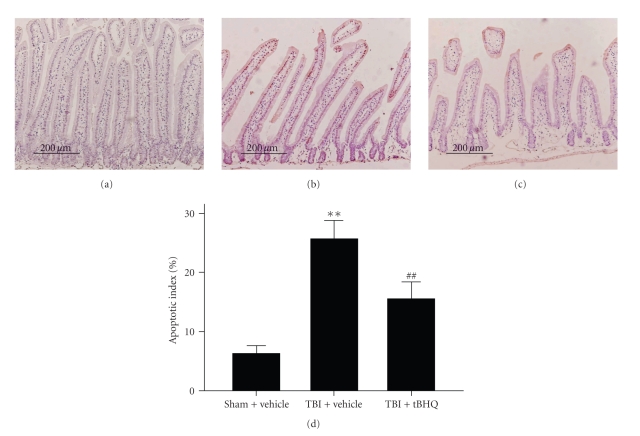
TUNEL immunohistochemistry staining in the intestinal tissue in sham + vehicle group, TBI + vehicle group, and TBI + tBHQ group (*n* = 6). (a) sham + vehicle group mice showing few TUNEL positive cells (stained brown); (b) TBI + vehicle group mice showing more TUNEL positive cells; (c) TBI + tBHQ group mice showing less TUNEL positive cells than TBI + vehicle group; (d) tBHQ treatment significantly decreased the apoptotic index in the intestinal tissue following TBI. **P < .01 versus sham + vehicle group; ^##^ *P* < .01 versus TBI + vehicle group.

Low apoptotic index was found in the sham + vehicle group mice intestinal tissue, as shown in Figure 4. In TBI + vehicle group, the apoptotic index in the studied mice gut was found to be significantly increased compared with that in sham + vehicle group animals. In TBI + tBHQ group, the apoptotic index in the intestinal tissue was significantly decreased. The results suggested that tBHQ treatment could inhibit apoptotic cell death in the intestine and could potentially reduce the mucosal injury following TBI.

## 4. Discussion

This study revealed that the inflammatory-related factors including NF-*κ*B, proinflammatory cytokines, and ICAM-1 in the intestine were significantly upregulated following TBI and could be suppressed when treated with tBHQ. At the same time, treatment with tBHQ markedly reduced TBI-induced apoptotic cell death in the intestine. These findings reported here suggest for the first time that tBHQ administration can suppress the intestinal inflammation and reduce the mucosal injury following TBI.

To defend against exogenous toxins and injury, cells possess a large number of cytoprotective and detoxifying enzymes whose expression is rapidly increased in response to insults. Many of these genes contain a common promoter element called the antioxidant response element (ARE). Several transcription factors can bind to ARE; however, Nrf2 is the major one that binds to and activates the expression of these ARE-mediated gene products [19–21]. In recent researches, the central role of Nrf2 in cell survival has been well eatablished in vitro and in vivo [22, 23]. Importantly, numerous lines evidence generated in the recent years have implied the anti-inflammatory effect of Nrf2 in a variety of experimental models [24–27]. Our previous studies showed that in response to TBI, mice lacking Nrf2 exhibited increased intestinal inflammatory response and mucosal injury [6, 7]. These provide the evidence that Nrf2 plays an important role in the gut injury after TBI. Upregulation of Nrf2 may bring great benefit to the TBI-induced intestinal inflammatory damage.

Synthetic phenolic antioxidant tBHQ used widely as a food-additive antioxidant for humans has long been of interest as effective inducer of Nrf2 [14, 28, 29]. There is abundant evidence that tBHQ treatment increases Nrf2 activation and confers protection [14, 30]. In vitro studies have shown that, as a result of its action on Nrf2, tBHQ is effective in inducing ARE-dependent gene expression in cultured astrogytes and protecting neurons from oxidative injury [31]. Other in vivo studies have shown that the neuroprotective effect of tBHQ on oxidative stress and ischemic injury is lost in Nrf2-deficient mice [14, 15]. Additionally, the majority of tBHQ-induced genes in glia and neurons have been shown to be dependent on Nrf2 by microarray analyses [29]. In light of the findings described above, we hypothesize that activation of Nrf2 with tBHQ may represent an attractive target for combating TBI-induced intestinal inflammatory response and mucosal injury.

The intestinal inflammatory response following trauma is characterized by intestinal recruitments of neutrophils and monocytes through activation of NF-*κ*B, releasing many inflammatory cytokines, and upregulation of adhesion molecule, which has been implicated in the pathogenesis of TBI-induced gastrointestinal dysfunction [4]. NF-*κ*B is one of the most important proinflammatory modulators, which can be activated by lesion-induced oxidative stress, bacterial endotoxin, or cytokines and subsequently transactivate the expression of many cytokines and adhesion molecules [32]. Proinflammatory cytokines, including interleukins (ILs) and TNF-*α* released early after an inflammatory stimulus, can initiate the infiltration of inflammatory cells into the intestine by activating ICAM-1 and other adhesion molecules [33]. NF-*κ*B activation enhances the transcription of proinflammatory cytokines, and the cytokines are known to in turn activate NF-*κ*B [34]. The positive feedback is believed to serve to amplify inflammatory signals and exacerbate tissue injury. Our study showed that at 24 h after TBI, upregulation of NF-*κ*B, TNF-*α*, IL-1*β*, and IL-6 was evident in the intestine and could be inhibited when treated with tBHQ. These results illustrated that tBHQ administration could suppress the intestinal inflammation following TBI.

The major changes of gastrointestinal function after TBI can be summarized into four aspects: gastrointestinal mucosa ischemia, gut motility dysfunction, disruption of gut barrier, and alteration of intestinal mucosal absorptive function. Apoptosis is an important factor in gastrointestinal physiological cell renewal, which can be triggered by noxious stimuli such as trauma and ischemia [35, 36]. Previous studies of our laboratory definitely demonstrated that marked apoptosis of the intestinal epithelial cells occurred after TBI, and it was supposed to play an important role in the gut barrier damage and increased permeability of the intestinal epithelium, leading to possible translocation of intraluminal microbes and bacterial toxins [5, 6]. However, there is still no effective treatment for the gut mucosa injury caused by TBI. In the present study, we found that at 24 h after TBI, increased intestinal mucosal apoptosis was evident and could be suppressed by tBHQ administration. These results suggested that tBHQ could afford protection to the TBI-induced gut mucosal damage. 

Although the precise mechanism regarding the anti-inflammatory ability of tBHQ remains elusive, several lines of evidence indicate that Nrf2 and NF-*κ*B signaling pathways contribute to the pathophysiological process. The prevailing theory is that Nrf2 interferes with inflammatory signaling pathways by inhibiting NF-*κ*B activation through the maintenance of cellular redox status. Oxidative stress from reactive oxygen species (ROS) is believed to be involved in the progression of gastrointestinal dysfunction secondary to TBI [37]. Activation of the NF-*κ*B signaling pathway has been shown to be responsive to excess ROS and is important in the generation of inflammation [26]. The antioxidant transcription factor Nrf2 has been shown to play an important role in limiting ROS levels and thereby affect redox-sensitive NF-*κ*B signaling pathway involved in the inflammation [19, 38]. It is therefore implied that tBHQ may play an important role in anti-inflammation by a mechanism of the augmentation of cellular antioxidative system via upregulation of Nrf2 signaling pathway resulting in decreased pro-inflammatory cytokines production and adhesion molecules expression via inactivation of NF-*κ*B signaling pathway. It is assured that further ingenious researches are needed and will be conducted in our laboratory.

In summary, the results showed that oral treatment with tBHQ prior to TBI markedly decreased NF-*κ*B activation, inflammatory cytokines production, and ICAM-1 expression in the gut. Additionally, tBHQ administration significantly attenuated TBI-induced intestinal mucosal apoptosis. These results suggest that tBHQ maybe an effective therapeutic drug for the treatment of TBI-induced gut injury with a potential mechanism of upregulation of Nrf2 signaling pathway.

## References

[1] Pilitsis JG, Rengachary SS (2001). Complications of head injury. *Neurological Research*.

[2] Hang CH, Shi JIX, Li JS, Wu W, Yin HX (2003). Alterations of intestinal mucosa structure and barrier function following traumatic brain injury in rats. *World Journal of Gastroenterology*.

[3] Doig CJ, Sutherland LR, Sandham JD, Fick GH, Verhoef M, Meddings JB (1998). Increased intestinal permeability is associated with the development of multiple organ dysfunction syndrome in critically ill ICU patients. *American Journal of Respiratory and Critical Care Medicine*.

[4] Hang CH, Shi JIX, Li JS, Li WQ, Wu W (2005). Expressions of intestinal NF-*κ*B, TNF-*α*, and IL-6 following traumatic brain injury in rats. *Journal of Surgical Research*.

[5] Hang CH, Shi JIX, Li JS, Li WQ, Yin HX (2005). Up-regulation of intestinal nuclear factor kappa B and intercellular adhesion molecule-1 following traumatic brain injury in rats. *World Journal of Gastroenterology*.

[6] Jin W, Wang H, Ji Y (2008). Increased intestinal inflammatory response and gut barrier dysfunction in Nrf2-deficient mice after traumatic brain injury. *Cytokine*.

[7] Jin W, Wang HD, Hu ZG, Yan W, Chen G, Yin HX (2009). Transcription factor Nrf2 plays a pivotal role in protection against traumatic brain injury-induced acute intestinal mucosal injury in mice. *Journal of Surgical Research*.

[8] Munday R, Munday CM (2004). Induction of phase II enzymes by 3H-1,2-dithiole-3-thione: dose-response study in rats. *Carcinogenesis*.

[9] Koh K, Cha Y, Kim S, Kim J (2009). tBHQ inhibits LPS-induced microglial activation via Nrf2-mediated suppression of p38 phosphorylation. *Biochemical and Biophysical Research Communications*.

[10] Nishizono S, Hayami T, Ikeda I, Imaizumi K (2000). Protection against the diabetogenic effect of feeding tert-butylhydroquinone to rats prior to the administration of streptozotocin. *Bioscience, Biotechnology and Biochemistry*.

[11] Lamb JG, Franklin MR (2000). Early events in the induction of rat hepatic UDP-glucuronosyltransferases, glutathione S-transferase, and microsomal epoxide hydrolase by 1,7-phenanthroline: comparison with oltipraz, tert-butyl-4-hydroxyanisole, and tert-butylhydroquinone. *Drug Metabolism and Disposition*.

[12] Nakamura Y, Kumagai T, Yoshida C (2003). Pivotal role of electrophilicity in glutathione S-transferase induction by tert-butylhydroquinone. *Biochemistry*.

[13] Münzel PA, Schmohl S, Buckler F (2003). Contribution of the Ah receptor to the phenolic antioxidant-mediated expression of human and rat UDP-glucuronosyltransferase UGT1A6 in Caco-2 and rat hepatoma 5L cells. *Biochemical Pharmacology*.

[14] Li J, Johnson D, Calkins M, Wright L, Svendsen C, Johnson J (2005). Stabilization of Nrf2 by tBHQ confers protection against oxidative stress-induced cell death in human neural stem cells. *Toxicological Sciences*.

[15] Shih AY, Li P, Murphy TH (2005). A small-molecule-inducible Nrf2-mediated antioxidant response provides effective prophylaxis against cerebral ischemia in vivo. *Journal of Neuroscience*.

[16] Shih AY, Imbeault S, Barakauskas V (2005). Induction of the Nrf2-driven antioxidant response confers neuroprotection during mitochondrial stress in vivo. *Journal of Biological Chemistry*.

[17] Hall ED (1985). High-dose glucocorticoid treatment improves neurological recovery in head-injured mice. *Journal of Neurosurgery*.

[18] Jin W, Wang H, Yan W (2009). Role of Nrf2 in protection against traumatic brain injury in mice. *Journal of Neurotrauma*.

[19] Itoh K, Chiba T, Takahashi S (1997). An Nrf2/small Maf heterodimer mediates the induction of phase II detoxifying enzyme genes through antioxidant response elements. *Biochemical and Biophysical Research Communications*.

[20] Motohashi H, Yamamoto M (2004). Nrf2-Keap1 defines a physiologically important stress response mechanism. *Trends in Molecular Medicine*.

[21] Osburn WO, Wakabayashi N, Misra V (2006). Nrf2 regulates an adaptive response protecting against oxidative damage following diquat-mediated formation of superoxide anion. *Archives of Biochemistry and Biophysics*.

[22] Lee JM, Calkins MJ, Chan K, Kan YW, Johnson JA (2003). Identification of the NF-E2-related factor-2-dependent genes conferring protection against oxidative stress in primary cortical astrocytes using oligonucleotide microarray analysis. *Journal of Biological Chemistry*.

[23] Calkins MJ, Jakel RJ, Johnson DA, Chan K, Yuen WK, Johnson JA (2005). Protection from mitochondrial complex II inhibition in vitro and in vivo by Nrf2-mediated transcription. *Proceedings of the National Academy of Sciences of the United States of America*.

[24] Rangasamy T, Guo J, Mitzner WA (2005). Disruption of Nrf2 enhances susceptibility to severe airway inflammation and asthma in mice. *Journal of Experimental Medicine*.

[25] Rangasamy T, Cho CY, Thimmulappa RK (2004). Genetic ablation of Nrf2 enhances susceptibility to cigarette smoke-induced emphysema in mice. *Journal of Clinical Investigation*.

[26] Thimmulappa RK, Lee H, Rangasamy T (2006). Nrf2 is a critical regulator of the innate immune response and survival during experimental sepsis. *Journal of Clinical Investigation*.

[27] Khor TO, Huang MT, Kwon KIH, Chan JY, Reddy BS, Kong AHN (2006). Nrf2-deficient mice have an increased susceptibility to dextran sulfate sodium-induced colitis. *Cancer Research*.

[28] (1999). Evaluation of certain food additives and contaminants. Forty-ninth report of the Joint FAO/WHO Expert Committee on Food Additives. *World Health Organization Technical Report Series*.

[29] Kraft AD, Johnson DA, Johnson JA (2004). Nuclear factor E2-related factor 2-dependent antioxidant response element activation by tert-butylhydroquinone and sulforaphane occurring preferentially in astrocytes conditions neurons against oxidative insult. *Journal of Neuroscience*.

[30] Yan D, Dong J, Sulik KK, Chen SY (2010). Induction of the Nrf2-driven antioxidant response by tert-butylhydroquinone prevents ethanol-induced apoptosis in cranial neural crest cells. *Biochemical Pharmacology*.

[31] Johnson DA, Andrews GK, Xu W, Johnson JA (2002). Activation of the antioxidant response element in primary cortical neuronal cultures derived from transgenic reporter mice. *Journal of Neurochemistry*.

[32] Chen F, Castranova V, Shi X, Demers LM (1999). New insights into the role of nuclear factor-*κ*B, a ubiquitous transcription factor in the initiation of diseases. *Clinical Chemistry*.

[33] Hang CH, Shi JIX, Tian J, Li JS, Wu W, Yin HX (2004). Effect of systemic LPS injection on cortical NF-*κ*B activity and inflammatory response following traumatic brain injury in rats. *Brain Research*.

[34] Neurath MF, Pettersson S, Meyer Zum Buschenfelde KH, Strober W (1996). Local administration of antisense phosphorothioate oligonucleotides to the p65 subunit of NF-*κ*B abrogates established experimental colitis in mice. *Nature Medicine*.

[35] Potten CS, Wilson JW, Booth C (1997). Regulation and significance of apoptosis in the stem cells of the gastrointestinal epithelium. *Stem Cells*.

[36] Zhang C, Sheng ZY, Hu S, Gao JC, Yu S, Liu YI (2002). The influence of apoptosis of mucosal epithelial cells on intestinal barrier integrity after scald in rats. *Burns*.

[37] Shohami E, Gati I, Beit-Yannai E, Trembovler V, Kohen R (1999). Closed head injury in the rat induces whole body oxidative stress: overall reducing antioxidant profile. *Journal of Neurotrauma*.

[38] Lee JM, Johnson JA (2004). An important role of Nrf2-ARE pathway in the cellular defense mechanism. *Journal of Biochemistry and Molecular Biology*.

